# Analyzing the coupling coordination between aviation logistics and the regional economy: Identifying coupling mechanisms and critical influencing factors

**DOI:** 10.1371/journal.pone.0323111

**Published:** 2025-05-09

**Authors:** Lijun Liang, Peirong Chen, Mengwan Zhang

**Affiliations:** 1 School of Business, Beijing Information Science and Technology University (BISTU), Beijing, China; 2 School of Management Science and Engineering, Beijing Information Science and Technology University (BISTU), Beijing, China; 3 School of Economics and Management, Beijing Forestry University, Beijing, China; Air Force Engineering University, CHINA

## Abstract

The relationship between aviation logistics and the regional economy (AL&RE) is mutually reinforcing. This study aims to develop a research framework for evaluating the coupling coordination level and identifying critical influencing factors in regional AL&RE by integrating the coupling coordination degree model with grey relational analysis. The framework is designed to guide regional policy formulation, fostering economic development through the coordinated advancement of AL&RE. To validate its effectiveness, an empirical analysis is conducted using data from Sichuan Province in China for the period 2013–2023. The key findings are as follows: (1) In Sichuan Province, local government expenditures on civil aviation development funds play a crucial role in supporting aviation logistics, while GDP and the proportion of investment in environmental pollution control relative to GDP significantly impact the regional economy. (2) The coupling degree between AL&RE remains high, with coordination generally improving, although occasional declines occur due to internal imbalances caused by external factors. (3) The influence of each indicator on coupling coordination varies, with civil aviation cargo turnover, throughput, and civil aviation take-off and landing sorties being the most significant. (4) Critical factors influencing AL&RE development differ from those affecting their coupling coordination, underscoring the nonlinear nature of this composite system. The study theoretically constructs a composite system for AL&RE and analyzes their coupling mechanism, addressing a significant research gap. It also integrates green indicators into the regional economic subsystem, offering a novel perspective for future research. Practically, the study provides recommendations for enhancing AL&RE coordination in Sichuan.

## Introduction

As the economy shifts towards a phase of high-quality development, emerging business models like cross-border e-commerce and cold chain logistics have seen substantial growth [1]. Aviation logistics, known for its safety, speed, and extensive reach, has become a vital driver of both social progress and economic prosperity [2]. This role was particularly significant during the COVID-19 pandemic. Specifically, since the crisis began, aviation logistics has been essential in mitigating the pandemic’s impact and ensuring supply chain stability, thanks to its rapid delivery capabilities and global mobility [3].

The level of regional economic vitality not only influences the demand for aviation logistics services but also affects the supply capacity of these services. A robust regional economy indicates a large market and heightened demand, thereby improving growth prospects and profitability within the aviation logistics sector. Furthermore, aviation logistics necessitates substantial investment in airport infrastructure, cargo airline operations, and associated services. A deficiency in economic strength at the regional level can impede the development and operation of essential aviation logistics infrastructure, thereby hindering sectoral growth. This challenge is particularly pronounced during the post-COVID-19 recovery phase, where pressure on aviation logistics infrastructure costs is anticipated to rise [4].

This analysis highlights the crucial interdependence between aviation logistics and the regional economy (AL&RE). Effective coordination between these systems can provide additional momentum for their development, creating a mutually reinforcing effect that neither system could achieve alone. However, inadequate coordination may impede their mutual promotion, and resources invested in the field may not fully enhance the system’s development due to limitations imposed by the other system. In the context of the mutual interaction between AL&RE, this study aims to address the following key research questions to advance the coordinated development of AL&RE: What is the nature of the coupling mechanism between AL&RE? How can the development status of both systems, as well as the coupling and coordination between them, be evaluated? What factors influence their coupling coordination? Finally, how should limited resources be allocated to the key areas that can generate the greatest developmental impact to promote the coordinated development of AL&RE?

To address the above question, this study develops a framework to assist regional managers and policymakers in evaluating the development levels and coordination between AL&RE, while identifying the critical factors influencing their coordination, considering regional differences. The main content and innovations of this study are as follows: (1) Theoretical innovation: Grounded in system theory, the study constructs a composite system of AL&RE and thoroughly analyzes its coupling mechanism. This fills the gaps in previous research where the coupling mechanism was neglected, providing theoretical support for future studies. (2) Innovation in measurement indicator systems: The study establishes indicator systems to assess the development levels of both subsystems. In creating the regional economic subsystem measurement system, the study integrates green economy indicators in line with the commitment to green development, offering a fresh perspective for future research. (3) Methodological innovation: The study introduces a Coupling Coordination Degree Model (CCDM) to evaluate the level of coordination development. Additionally, to investigate critical factors influencing coordination, Grey Relational Analysis (GRA) is utilized to quantify the relationship between subsystem measurement indicators and system coupling coordination. This dual approach enables a deeper analysis of the interactions within the system and helps identify areas for improvement in the coordination process. (4) Policy Implications: Using Sichuan Province as an empirical case, the study validates the framework with data from Sichuan Province (2013–2023). Furthermore, based on the findings from Sichuan, the study offers policy recommendations for effectively allocating resources and promoting efficient regional economic growth.

In the remainder of this part, a literature review is presented. Part 2 details the construction of the composite system and the analysis of its coupling mechanism. Part 3 describes the research materials and methods, including the development of measurement indicator systems for the AL&RE, formulation of entropy value method equations, development of the CCDM, the formulation of the GRA equations, the selection of the empirical region, and the collection of data. Part 4 presents the research results and discussion, encompassing the analysis of indicator weights, coupling coordination degree, GRA results, and policy recommendations. Part 5 concludes the study, addressing its limitations and suggesting directions for future research.

## Literature review

### The causality between AL&RE

Given the increasing significance of aviation logistics in regional economic development and the interactive relationship between AL&RE, numerous studies have quantitatively examined the causality between AL&RE, seeking to validate and enhance the understanding of this relationship. For instance, Zhang and Graham [5] conducted quantitative research, discovering that a reciprocal causal relationship is more likely to prevail in underdeveloped economies. Conversely, in more developed economies, the causality typically flows in one direction, from aviation logistics to economic growth.

In light of the regional disparities, researchers have undertaken studies concentrating on specific regions, economic organizations, and countries. Tolcha et al. [6] employed vector autoregression models to identify both short-term and long-term causalities between AL&RE in South Africa. Their results revealed heterogeneous causality: in the long run, for South Africa, Nigeria, and Kenya, the causal direction flows from economic development to aviation logistics, while for Ethiopia, aviation logistics fosters economic development. Law et al. [7] investigated the relationship between AL&RE in the CLMV countries of Southeast Asia (Cambodia, Laos, Myanmar, and Vietnam), discovering a bidirectional causal relationship in the long term. Nguyen [8] conducted Granger causality tests to examine the relationship between AL&RE in Asia. The study confirmed a bidirectional causality in most regions, while in South Asia, a unidirectional causality was identified from economic growth to aviation passenger transport, and in Central and West Asia, from economic growth to aviation freight. Similarly, Ali [9] utilized aviation passenger numbers and freight volumes as indicators of aviation logistics, employing econometric techniques for causal analysis. Their findings suggest a one-way short-run causal relationship from aviation logistics to economic growth in the BRICS countries.

These studies indicate that the direction of causality and the time lags (short-term or long-term) may vary across regions due to differences in spatial, economic, cultural, and social characteristics. These findings provide a preliminary understanding of the relationship between AL&RE.

### The interaction and critical factors of AL&RE

As the causality between AL&RE has been studied to some extent, academic focus is now shifting towards identifying the critical factors that mediate and drive this interaction. Aviation logistics plays a crucial role in facilitating the global movement of passengers and goods [10]. Previous studies have primarily explored the interaction between AL&RE from these two perspectives or by combining both, with an emphasis on the critical interrelated factors.

The role of aviation logistics in driving regional economic development has been extensively studied. Concerning the impact of aviation passenger transport on the regional economy, Adedoyin et al. [11] employed econometric methods to examine the relationship between aviation passenger transport and GDP per capita. Their findings indicated that growth in aviation passenger traffic significantly enhances GDP per capita, leading to the formulation of the Air Transport-Led Growth Hypothesis (ALGH). Raihan et al. [12] applied the Autoregressive Distributed Lag (ARDL) model to Malaysian data, validating this hypothesis and demonstrating that aviation passenger transport significantly impacts both short-term and long-term GDP growth in Malaysia. Regarding the effect of aviation cargo on the regional economy, Zhou et al. [13] utilized panel data regression analysis to study China’s aviation cargo industry, revealing that growth in aviation cargo leads to increased regional employment. Considering both passenger and cargo transport perspectives within aviation logistics, Sherd [14] and Njoya et al. [15], through computable model analysis, found that aviation logistics contributes to economic output, income, employment, population size, and GDP in the regions under study.

The influence of the regional economy on aviation logistics has also been extensively examined. From the perspective of aviation passenger transport, Albayrak et al. [16] conducted a literature review and identified economic factors affecting aviation passenger traffic, such as population, income, and regional economic structure. İNAN et al. [17] through multiple linear regression analysis, found that GDP and total population significantly impact the number of aviation passengers. Subsequently, Bai and Wu [18] revealed a bidirectional relationship between aviation passenger throughput and GDP in Jiangsu Province, employed a VAR model and Granger causality tests. Regarding freight, Li et al. [19] applied network theory to demonstrate that China’s aviation freight is significantly influenced by GDP and industrial structure. In a comprehensive study of both passenger and cargo transport, Hakim and Merkert [20] employed a fixed effects model and a three-step error correction mechanism (ECM) to examine the determinants of aviation logistics demand in South Asia. The study found that per capita income, investment, and flight frequency significantly influence passenger demand, while per capita income, investment, and industrial structure are key drivers of aviation freight demand.

These studies demonstrate the multifaceted and mutually reinforcing nature of the interaction between AL&RE. Research has highlighted the significant role of aviation logistics in fostering economic growth, particularly through aviation passenger transport and aviation cargo. Moreover economic factors such as GDP, population size, income levels, and industrial structure have been consistently identified as key drivers of both aviation passenger and cargo transport. Furthermore, the varying effects and critical factors observed in different regional contexts highlight the complexity of this interaction.

### Measurement of coupling relationship between AL&RE

Due to the significant causal relationship and interconnection between AL&RE, it is likely that a certain degree of coupling exists between them, marked by their interdependence in coordination and complementarity. Therefore, investigating the coupling and coordination between the two is of great importance. Some scholars have already focused on this issue and analyzed the coupling and coordination relationship between AL&RE.

Regarding the study of the coupling and coordination relationship between AL&RE, Yan et al. [21] assessed the high-quality development of the AL&RE industries using principal component analysis and the Slacks-Based Measure (SBM) model, respectively, and subsequently evaluated their synergy through coupling coordination degree (CCD) analysis. The results revealed considerable regional variations in CCD across China. Similarly, Du et al. [22] utilized the CCDM and the Entropy Method (EM) to quantitatively investigate the coupling coordination and spatial distribution of AL&RE across 30 provinces in China. Their findings indicated that the CCD between China’s AL&RE is shifting from a state of discoordination to a higher level of coordination, although notable imbalances in CCD levels persist across the studied regions. An et al. [23] applied the theory of regional coordinated development and coupling theory, employing the CCDM to analyze data from 31 provinces and municipalities in China. They also identified significant regional disparities in economic development, logistics development, and the CCD across China.

The methods employed in previous studies are similar, yielding consistent results. These studies generally utilize computational models to assess the development levels of AL&RE systems, followed by the construction of a CCDM to measure the coupling degree (CD) and CCD. However, these studies primarily focus on evaluating the coupling and coordination between AL&RE, often overlooking the complex coupling mechanisms underlying this relationship. Moreover, after the evaluation, policy recommendations are typically suggested from a subjective, qualitative standpoint, lacking an identification of the critical factors influencing their coordination.

Overall, the literature review highlights that the causal relationship and coupling coordination between AL&RE vary significantly across regions. Their interaction is influenced by multiple factors, often resulting in positive mutual reinforcement. However, current research mainly focuses on measuring the coupling and coordination levels between AL&RE, with insufficient analysis of the complex coupling mechanisms behind their interaction, which is shaped by multiple interwoven factors. Additionally, there is a lack of exploration into the critical factors influencing coordination. Furthermore, while empirical studies often analyze case studies of different regions, they tend to focus on the current status and future development of those areas, without offering a universally applicable analytical framework or strategic guidance for various regions.

This study addresses existing research gaps by developing a framework that identifies the coordinated development level of regional AL&RE and its critical influencing factors, assisting regional managers in adjusting policies based on actual conditions. Specifically, the study employs systems theory to create an AL&RE composite system and analyze its coupling mechanism. Based on this theoretical foundation, the study develops measurement indicator systems and a CCDM to examine the coupled and coordinated development of AL&RE. In alignment with green development principles, the study incorporates green economy elements as key indicators for assessing regional economic progress. Additionally, the study combines GRA to identify critical factors affecting the coupling coordination between AL&RE. Through the integration of these two methods, the study provides a comprehensive understanding of the complex process of coupling between AL&RE and explores key pathways to enhance the coupling coordination process. Finally, the framework’s feasibility is empirically validated through a case study of Sichuan Province, accompanied by policy recommendations based on the findings.

## Composite system construction and coupling mechanism analysis

### Composite system construction

Systems theory posits that phenomena should be analyzed as integrated wholes, highlighting the significance of interactions among elements to understand the organization, functioning, and outcomes of an entity [24]. Consequently, this study examines AL&RE from a systems perspective and develops a composite system that illustrates their interdependent interactions.

### Aviation logistics system.

 Aviation logistics encompasses the entire process of transporting goods and passengers from one location to another via air transport [25]. This process can be systematically analyzed and categorized through the lens of supply and demand relationships. On the supply side, it includes various aspects of logistics services, such as transport efficiency, human resources, network coverage, and supporting infrastructure [26]. On the demand side, it primarily reflects the market demand for goods and passenger transportation services [27].

By employing a supply-demand framework to analyze aviation logistics, we gain valuable insights into the characteristics, structure, and components of the aviation logistics system. Understanding the interplay between these two sides is essential for optimizing resource allocation, enhancing service quality, and fostering sustainable growth within the aviation logistics sector.

### Regional economic system.

 The regional economy refers to the organization, development, and operation of economic activities within a specific geographical area [28]. Drawing from previous studies on the internal factors of the regional economy [29,30], this research systematically categorizes the regional economy into multiple dimensions, focusing on the following aspects:

(1) Overall economic performance: This serves as a crucial foundation for regional economic vitality and is a prerequisite for other economic indicators, with core metrics such as Gross Domestic Product (GDP).
(2) Economic structure: The economic structure reflects the contributions of various industries and sectors to the regional economy, typically divided into primary (agriculture), secondary (industry), and tertiary (services) sectors. Among these sectors, the share of the tertiary sector partially reflects the degree of optimization and upgrading of the economic structure, making it a vital variable for analysis. Since this study emphasizes the coupling coordination between AL&RE, it considers the proportion of the tertiary sector as an important factor.
(3) Trade activity intensity and economic openness: These factors reflect the internal consumption dynamics within a region and its exchange relationships with external markets. Both are crucial in driving regional economic development.
(4) Sustainable development: Sustainable development is vital for the long-term health of the regional economy. In line with global commitments under the UN framework for the 2030 Agenda for Sustainable Development, this study seeks to achieve balanced growth across economic, social, and environmental dimensions to ensure collective progress [31]. Consequently, this study integrates green sustainability as a fundamental consideration in regional economic development.

This study offers a comprehensive understanding of the current state and developmental trajectory of the regional economy through a thorough analysis of these dimensions, providing robust support for policymaking, resource allocation, and strategic planning.

### AL&RE composite system framework

AL&RE are two distinct systems, each with its own unique structures. However, their internal factors and the relationship between the two systems are closely interwoven. Based on the definition that a composite system consists of two or more interrelated and interdependent subsystems [32], this study conceptualizes AL&RE as two subsystems within the composite system.

**Fig 1:**
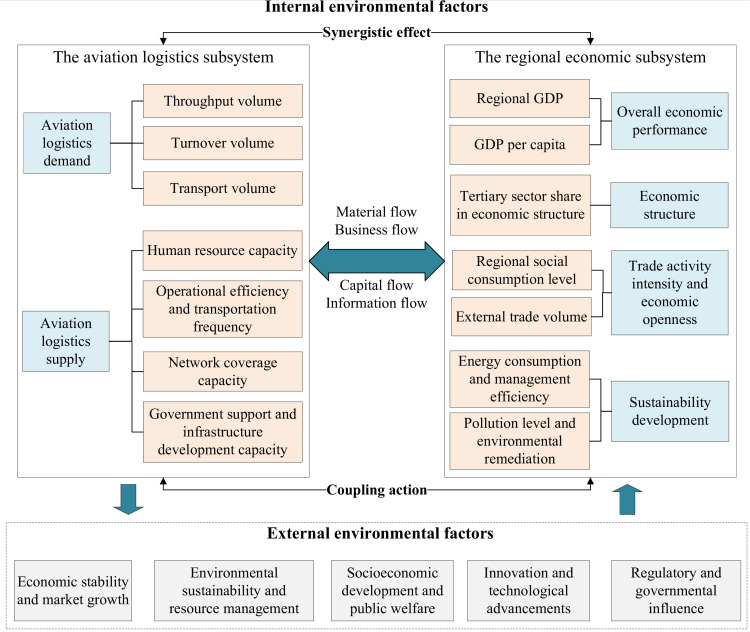
AL&RE composite system framework.

Specifically, the elements of aviation logistics and various factors of the regional economy are interconnected through complex interactions, forming an input-output mechanism. This mechanism facilitates the transmission and circulation of elements such as capital and information between the subsystems and their internal components through material exchanges and business flows, thereby linking the aviation logistics subsystem with the regional economic subsystem into a cohesive whole. Furthermore, both AL&RE are influenced by and, in turn, exert reciprocal effects on various environmental factors, including economic, ecological, social, technological, and political elements. Based on these complex interrelationships, this study develops the AL&RE composite system. The framework is illustrated in Fig 1.

### Coupling mechanism analysis of AL&RE

Building on the established AL&RE composite system, this study further investigates the coupling mechanism between the AL&RE subsystems and their internal factors, thereby providing a theoretical framework for subsequent research.

### The significance of aviation logistics in driving regional economic growth

Aviation logistics play a crucial role in the modern economic system. The throughput, turnover, and transportation volumes in aviation logistics reflect its demand structure and multidimensional characteristics in scale, frequency, and volume. Enhancing its supply capacity, particularly in areas such as human resources, transport efficiency, network capacity, and government policies, significantly impacts various aspects of the regional economy. Specifically, the contribution of aviation logistics to regional economic development can be illustrated in the following ways:

First, the growing demand for aviation logistics activities directly generates economic benefits, thereby boosting overall economic output, which in turn supports higher levels of GDP and per capita GDP. Moreover, aviation logistics, as a fast and secure mode of transportation, is primarily utilized for the delivery of high-value products, which are often categorized within the tertiary sector. Consequently, the increased demand for aviation logistics will significantly promote the development of the tertiary sector, ultimately facilitating the optimization of the regional economic structure. In addition, aviation logistics plays a key role in influencing trade activity intensity and economic openness by enhancing the movement of goods and services within regions and across borders. This increased trade flow supports regional integration into global supply chains, driving further economic growth. Finally, the revenue generated by the aviation logistics industry contributes to the green development of the regional economy by providing funding and vitality for initiatives focused on sustainability.

Improvements in aviation logistics capacity from the supply side can significantly enhance the overall efficiency of supply chains, directly contributing to regional GDP growth and establishing a robust economic foundation. Moreover, the expansion of supply capacity enables more efficient and reliable transportation of high-value products in the tertiary sector, further promoting the rapid development of these industries and accelerating the optimization and upgrading of industrial structures. Additionally, enhanced supply capabilities in aviation logistics strengthen a region’s competitive position in the global market, facilitating smoother trade exchanges between regions and countries. The expansion of supply capacity often aligns with the optimization of transportation efficiency and systems, which includes the introduction of new transportation methods and low-emission technologies. This, in turn, drives the harmonious development of the regional economy and environmental sustainability, fostering long-term economic viability.

In conclusion, aviation logistics, through comprehensive enhancements in both demand and supply capabilities, has emerged as a crucial driver of regional economic growth, the facilitation of global market interactions, and the promotion of environmental sustainability.

### The reverse impact of regional economic growth on aviation logistics development

Various factors of the regional economy—including overall economic performance, economic structure, trade activity intensity, economic openness, and sustainable development—significantly influence both the demand and supply of aviation logistics. A detailed analysis of this mechanism is presented below:

Firstly, high GDP and per capita GDP typically indicate elevated consumption levels and purchasing power within a region, which directly drives the demand for aviation logistics. Moreover, the development of the tertiary sector often relies on aviation logistics to ensure the timely delivery of products, particularly in high-tech, pharmaceutical, and luxury goods industries. Therefore, the growth of the tertiary sector directly contributes to the increased demand for aviation logistics services. Additionally, the frequency of social consumption and international trade activities correlates with a heightened demand for the transportation of goods, which accelerates the growth of aviation logistics demand. Finally, in regions with high energy efficiency and pollution control levels, aviation logistics services often depend on low-carbon, environmentally friendly technologies and methods. This trend may lead to increased demand for aviation logistics due to consumers’ potential preference for green consumption.

On the supply side, as the economy evolves, businesses increasingly seek efficient and timely logistics services. Aviation logistics, recognized as a rapid and effective mode of transportation, will be driven by this growing demand to enhance and expand supply-side capabilities to meet rising needs. Furthermore, the expansion of the tertiary sector directly boosts the demand for improved transportation efficiency and reliability in aviation logistics. In response, the aviation logistics industry continually innovates and refines its transportation and supply chain management systems, thereby enhancing supply-side capabilities in terms of technology, infrastructure, and service quality. Additionally, as regional and international trade progresses and as goods and markets diversify, the supply side of aviation logistics must adapt to new trade requirements. This will necessitate the sector to increase transportation capacity, broaden flight routes, modernize transportation methods, and implement advanced technologies to manage cross-border logistics tasks. Finally, the supply side also benefits from the application of green technologies, including the use of low-emission aircraft, energy-efficient shipping facilities, and sustainable operational management, which promote the long-term sustainability of the aviation logistics industry.

In summary, regional economic growth significantly influences both the demand and supply of aviation logistics development.

### Coupling mechanism analysis

In physics, the term “coupling” refers to the phenomenon where two or more systems synchronize through interaction and mutual influence [33]. The development of the AL&RE creates a mutually reinforcing and interdependent coupling system. Within this system, AL&RE interact through multidimensional mechanisms such as material and business flows, facilitating the transfer and circulation of capital, information, and other critical elements among internal factors. This coupling relationship exhibits a bidirectional feedback effect, where aviation logistics, as a vital transport industry and service system, significantly influences the dynamic changes within the regional economy, while the structural evolution of the regional economy directly impacts the development trajectory of the demand and supply capacity of aviation logistics.

Furthermore, the coupling system between AL&RE extends beyond internal interactions to encompass external environmental factors, such as economic, ecological, social, technological, and political elements. These factors interact with and influence the internal environment of the composite system, affecting the layout, development trajectory, and overall efficiency of both AL&RE at various levels. The transmission and exchange of these external factors within the system further propel the coupling and collaborative development between AL&RE.

In this coupling mechanism, the optimal state is reached when “coordination” is established. The coupling coordination of the AL&RE composite system is primarily manifested through information sharing, resource balance allocation, complementarity, and symbiosis among the internal subsystems. To achieve efficient regional economic development, it is crucial to adopt a systematic, holistic perspective that emphasizes the coordinated development of AL&RE. Concentrating solely on the advancement of one aspect often leads to imbalances in the internal development of the system, which in turn restricts overall effectiveness and may even result in resource waste and inefficiency. By analyzing the level of coordination development and the influencing factors within the AL&RE system, scientific policies can be formulated to ensure efficient resource allocation and optimization, thereby promoting the coordinated and efficient development of the composite system.

## Materials and methods

**Fig 2:**
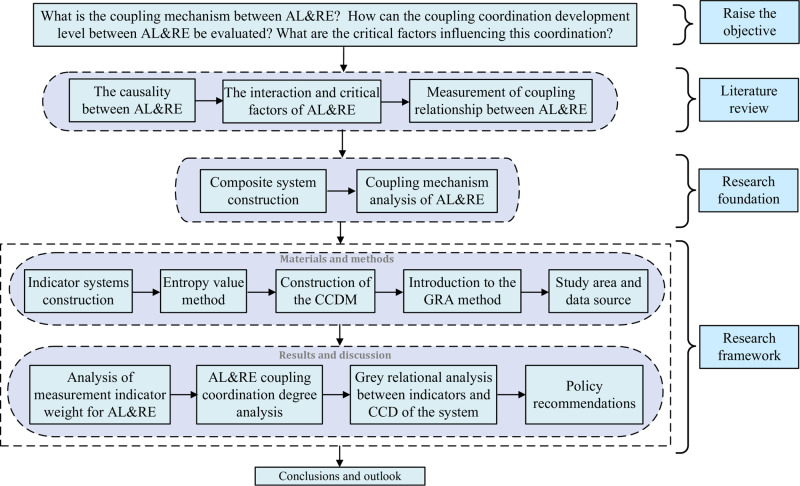
The research roadmap of this study.

Building on the construction of the composite system and the analysis of the coupling mechanism, this study aims to establish a research framework for evaluating and analyzing the coupling coordination level of AL&RE. It also seeks to identify critical influencing factors, thereby providing policymakers with a foundation and guidance for decision-making. To achieve this, measurement indicator systems are developed to assess AL&RE development, and a CCDM is constructed to evaluate their coupling and coordination. By integrating the CCDM with GRA, the study identifies the key factors affecting the coupling coordination of the system. Finally, to validate the applicability of the framework, an empirical analysis is conducted using Sichuan Province as a case study. The research roadmap for this study is illustrated in Fig 2.

### Indicator systems construction

Building on the established AL&RE subsystems and their coupling mechanism analysis, this study develops measurement indicator systems for the AL&RE subsystems, taking into account principles such as complexity, reliability, and sustainability in the selection of indicators.

#### Construction of the aviation logistics indicator system.

This study provides a comprehensive evaluation of the aviation logistics subsystem from both demand and supply perspectives. The selection of demand indicators is primarily based on the overall scale, frequency, and volume of transportation, while supply indicators aim to reflect the level of human resources, transport efficiency, network coverage capability, and government support for infrastructure development.

Notably, each take-off and landing process in aviation logistics entails a certain time investment, including flight preparation, take-off, cruising, and landing. An increase in civil aviation take-off and landing sorties generally indicates that more transportation tasks are completed within a given timeframe, thereby enhancing transport efficiency per unit of time. Additionally, the growth in flight route miles signifies the expansion of the aviation network, improving regional connections and services. Therefore, flight route miles are utilized to evaluate the coverage capacity of the aviation network. Furthermore, given the complexity of directly measuring infrastructure, this study posits that government funding is a crucial driver for infrastructure development [34]. Consequently, the local government expenditures on civil aviation development funds is chosen as an indicator reflecting government support and infrastructure development capacity.

Based on these considerations, the aviation logistics subsystem measurement indicator system is constructed, as illustrated in Table 1.

**Table 1:** The measurement indicator system for the aviation logistics subsystem.

Subsystem	Primary indicators	Secondary indicators
The aviation logistics subsystem	Aviation logistics demand	Civil aviation cargo throughput
		Civil aviation passenger throughput
		Cargo turnover of civil aviation
		Passenger turnover of civil aviation
		The volume of cargo transported by civil aviation
		The volume of passengers transported by civil aviation
	Aviation logistics supply	Number of employees in the aviation transport industry
		Civil aviation take-off and landing sorties
		Civil aviation flight route miles
		Local government expenditures on civil aviation development funds

### Construction of the regional economy indicator system

This study incorporates the objectives of green development, constructing a measurement indicator system for the regional economic subsystem based on four key economic dimensions, as detailed in Table 2.

**Table 2:** The measurement indicator system for the regional economic subsystem.

Subsystem	Primary indicators	Secondary indicators
The regional economic subsystem	Economic performance	Gross domestic product (GDP)
		Per capita national income
	Economic structure	Value added of tertiary industry as a proportion of GDP
	Trade intensity and economic openness	Total retail sales of consumer goods
		Total foreign trade imports
		Total foreign trade exports
	Green economy	Elasticity coefficient of energy consumption
		Elasticity coefficient of electricity consumption
		The proportion of investment in environmental pollution control relative to GDP

The elasticity coefficients of energy and electricity consumption illustrate the reliance of economic growth on these resources, with lower coefficients signifying more favorable conditions for green economic development. Given the essential role of electricity in both energy consumption and economic growth, these coefficients are introduced as indicators to evaluate the progress of the green economy. Additionally, the proportion of investment in environmental pollution control relative to GDP highlights the region’s dedication to promoting green economic development.

### Entropy value method

Entropy is a fundamental concept that quantifies the level of randomness or uncertainty inherent in a given event [35]. The specific operational steps are as follows:

Step 1: The range normalization method is employed to normalize both positive and negative indicators, ensuring consistency in dimensions across all indicators. The formula is as follows:

When the variable attribute is positive, the formula is as follows:


$$Y_{ij}=\frac{X_{ij}-{\min(X}_{j})}{\max(X_{j})-\min(X_{j})}$$ (1)

When the variable attribute is negative, the formula is as follows:


$$Y_{ij}=\frac{\max(X_{j})-X_{ij}}{\max(X_{j})-\min(X_{j})}$$ (2)

In the formula, ‘*i* *= 1, 2, …, m*’ represents the year, and ‘*j = 1, 2, …, n*’ denotes the number of measurement indicators. ‘*X*_*ij*_’ refers to the original data for the *j*-th indicator in the *i*-th year, while ‘*Y*_*ij*_’ represents the normalized value. ‘*min(X*_*j*_*)*’ and ‘*max(X*_*j*_*)*’ correspond to the minimum and maximum values of the *j*-th indicator, respectively. In this study, the elasticity coefficients of energy consumption and electricity consumption are treated as negative indicators, whereas the other indicators are considered positive.

Step 2: To calculate the entropy value ‘*P*_*ij*_*’*, which reflects the relative importance of the *j*-th indicator in the *i*-th year, the following formula is employed:


$$P_{ij}=\frac{X_{ij}}{\sum_{i=1}^{m}X_{ij}}$$ (3)

Step 3: To determine the information entropy weight of the indicator, denoted as ‘*e*_*j*_’, the formula is as follows:


$$e_{j}=-\frac{1}{lnm}\sum_{i=1}^{m}P_{ij}\ln\left(P_{ij}\right),0\leq e_{j}\leq 1$$ (4)

Step 4: To calculate the weight of the indicator, denoted as ‘*W*_*j*_’, the formula is as follows:


$$W_{j}=\frac{1-e_{j}}{\sum_{j=1}^{n}\left(1-e_{j}\right)},j=1,2,3,...,n$$ (5)

Step 5: To determine the comprehensive development level of the evaluation object, denoted as ‘*U*’, the following formula is employed:


$$U=\sum_{j=1}^{m}{W_{j}Y}_{ij}$$ (6)

### Construction of the coupling coordination model

The Coupling Coordination Degree Model (CCDM) is widely employed to assess the coordination of composite systems [36,37]. This study applies the CCDM to evaluate the coupling and coordination between AL&RE. The CCDM illustrates the transition from disorder to order in system design [38] and includes three essential metrics: the Coupling Degree (CD), Coordination Indicator (CI), and the Coupling Coordination Degree (CCD). The CD measures the heterogeneity of interactions and feedback among systems, indicating whether they converge or diverge. The CI evaluates the positive and negative impacts of inter-system interactions. The CCD is derived by integrating the CD and CI [39]. The construction of the CCDM consists of the following three steps:

Step 1: The general formula for calculating the CD is as follows:


$$C={\left[\frac{\prod_{i=1}^{n}U_{i}}{{\left(\frac{1}{n}\sum_{i=1}^{n}U_{i}\right)}^{n}}\right]}^{\frac{1}{n}}$$ (7)

In the formula, ‘*C*’ represents the CD between subsystems, reflecting the extent of interaction among them. The *C*-value ranges from 0 to 1, while ‘*n*’ indicates the number of coupled subsystems. This study concentrates on the coupling between the AL&RE subsystems, where *n* = 2, with ‘*U*_*1*_’ and ‘*U*_*2*_’ denoting the development levels of the AL&RE subsystems, respectively. Consequently, in this study, the CD model for the AL&RE subsystems is formulated as follows:


$$C={\left[\frac{U_{1}U_{2}}{{\left(\frac{U_{1}+U_{2}}{2}\right)}^{2}}\right]}^{\frac{1}{2}}$$ (8)

Step 2: The formulas for calculating the CI and CCD are expressed as follows:


$$T={aU}_{1}+{bU}_{2}$$ (9)

$$D=\sqrt{C\times T}$$ (10)

In these formulas, ‘*T*’ represents the CI, while ‘*D*’ denotes the CCD. The coefficients ‘*a*’ and ‘*b*’ remain undetermined, subject to the constraint that ‘*a*’ + ‘*b*’ = 1. According to existing research [40], both the AL&RE subsystems are assigned a coefficient value of 0.5.

Step 3: Drawing from previous research by relevant scholars [41,42], this study categorizes the CCD between the AL&RE subsystems into ten levels, as detailed in Table 3.

**Table 3:** Types and characteristics of CCD.

Degree of coupling coordination	Type of coupling coordination	Characteristic
D ∈[0.0,0.1)	Extreme dissonance	The improvement of the AL&RE composite system neglects the interaction between the two subsystems.
D ∈[0.1,0.2)	Severe dissonance	
D ∈[0.2,0.3)	Moderate dissonance	
D ∈[0.3,0.4)	Mildly dysfunctional	One subsystem predominates the AL&RE composite system development, while there are coordination issues with another subsystem.
D ∈[0.4,0.5)	Nearly dysfunctional	
D ∈[0.5,0.6)	Barely coordinated	The two subsystems in the AL&RE composite system development process can mutually enhance one another, resulting in a notable degree of coordinated development effect.
D ∈[0.6,0.7)	Elementary coordination	
D ∈[0.7,0.8)	Intermediate coordination	
D ∈[0.8,0.9)	Good coordination	The two subsystems in the AL&RE composite system development process have attained mutual coordination, and the AL&RE subsystems are experiencing synergistic and positive development.
D ∈[0.9,1.0)	Quality coordination	

### Grey relational analysis method

The Grey Relational Analysis (GRA) method is a data analysis technique employed to evaluate the relative importance of various features by evaluating them based on assigned ranks or scores. This process produces a significance sequence that identifies the features most influential to the phenomenon under investigation [43]. While the CCDM offers insights into the developmental stages of coupling and coordination among subsystems, it fails to capture the critical factors driving such cooperation. To address this limitation, this study presents the GRA model to identify the critical factors influencing the coordination of the AL&RE composite system. The relevant formulas are outlined as follows:

Step 1: To establish the analytical framework, the reference sequence, which represents the system’s behavioral traits, and the comparative sequence, which includes factors influencing the system’s behavior, are defined. The specific formulations for these sequences are as follows:


$$x_{0}=\left\{x_{0}(t)|t=1,2,...,m\right\}$$ (11)

$$x_{j}=\left\{x_{j}(t)|t=1,2,...,m\right\},j=1,2,...,n$$ (12)

In the formulas, ‘*x*_*0*_’ represents the reference sequence. This study examines the coupling coordination between the AL&RE subsystems, resulting in a single reference sequence. The variable ‘*t*’ denotes the year. ‘*x*_*0*_*(t)*’ represents the value of the evaluation object in the *t*-th year, which, in this context, refers to the CCD calculated for the *t*-th year.

‘*x*_*j*_’ represents the comparative sequence, with ‘*j*’ indicating the measurement indicators. ‘*x*_*j*_*(t)*’ denotes the value of the *j*-th indicator in the *t*-th year.

Step 2: Data de-dimensionalization is necessary due to the differing physical interpretations of each system factor, which may lead to varying data dimensions. To enable meaningful comparisons, dimensionless data processing is employed. In this study, dimensionless data were derived by dividing each value in the sequence by the mean.

Step 3: After identifying the sequences and conducting data de-dimensionalization, the grey relational coefficient is calculated using the following formula:


$$\delta_{j}(t)=\frac{\Delta \min+\rho \Delta \max}{\Delta j(t)+\rho \Delta \max}$$ (13)

In the formula, ‘Δj(t)’ denotes the absolute difference between the dimensionless value of indicator ‘*j*’ in the *t*-th year. ‘Δmin’ and ‘Δmax’ represent the minimum and maximum absolu*t*e differences between the evaluation object and the *j*-th indicator, respectively. ‘ρ’ indicates the resolution coefficient, which is established at 0.5 based on prior research [44].

Step 4: To determine the grey relational degree ‘*R*’ (0 < R < 1) in this study, where a larger ‘*R*’ signifies a stronger correlation between the coupling coordination of AL&RE and the measurement indicator. The calculation formula is as follows:


$$R=\frac{1}{m}\sum_{j=1}^{m}\delta_{j}(t)$$ (14)

### Study area and data source

This study uses Sichuan Province as an empirical case to test and analyze the proposed research framework. The selection of Sichuan is primarily based on the following considerations:

Firstly, Sichuan was chosen due to its strategic emphasis on aviation logistics, a key sector within the region. The “14th Five-Year Plan for Modern Logistics Development in Sichuan” (2021) outlines critical objectives, such as optimizing airport infrastructure, enhancing collaboration, and modernizing airport capacities. Given Sichuan’s focus on developing aviation logistics, this study aims to identify the coupling coordination level and the critical factors between the province’s AL&RE. The findings provide practical recommendations that align with the province’s current policy priorities and development focus, thereby enhancing the practical significance of the study.

Furthermore, Sichuan’s central-western location, with a high dependence on aviation logistics, fosters significant interactions between AL&RE. This close relationship often manifests as a strong coupling, making the exploration of the coupling coordination level highly valuable. Through the case of Sichuan Province, this study offers a deeper understanding of such a closely connected system and helps identify the critical factors influencing coordinated development.

This study utilizes foundational research data on AL&RE in Sichuan Province from 2013 to 2023. The selection of this period is based on the availability and relevance of data. The data sources include the Sichuan Statistical Yearbook, the Sichuan Provincial Government Fund Expenditure Final Accounts Table, the Sichuan Provincial Economic and Social Development Statistical Bulletin, and the CEIC Database. The specific raw data are provided in S1 Table and S2 Table.

## Results and discussion

### Analysis of measurement indicator weight for AL&RE

Indicator weights are crucial for evaluating the contribution of each indicator to the overall system, allowing managers to prioritize optimization efforts and allocate resources effectively. Employing the raw data from Sichuan Province and following the steps outlined in the EM, the measurement indicator weights are displayed in Table 4. The normalized indicator values derived during the calculation process can be found in S3 Table.

**Table 4:** Weights of measurement indicators.

Subsystem	Primary indicator	Secondary indicator	Weight
The aviation logistics subsystem	Aviation logistics demand	Civil aviation cargo throughput	0.0635
		Civil aviation passenger throughput	0.1046
		Cargo turnover of civil aviation	0.0789
		Passenger turnover of civil aviation	0.0906
		The volume of cargo transported by civil aviation	0.0807
		The volume of passengers transported by civil aviation	0.0904
	Aviation logistics supply	Number of employees in the aviation transport industry	0.1423
		Civil aviation take-off and landing sorties	0.0911
		Civil aviation flight route miles	0.0605
		Local government expenditures on civil aviation development funds	0.1975
The regional economic subsystem	Economic level	GDP	0.1383
		Per capita national income	0.1249
	Economic structure	Value added of tertiary industry as a proportion of GDP	0.0923
	Trade level	Total retail sales of consumer goods	0.1131
		Total foreign trade import	0.0990
		Total foreign trade exports	0.1197
	Green economy	Elasticity coefficient of energy consumption	0.0826
		Elasticity coefficient of electricity consumption	0.1011
		The proportion of investment in environmental pollution control relative to GDP	0.1289

As presented in Table 4, the results reveal a notable discrepancy in the weight values of the indicators across their respective subsystems, indicating that each factor has different marginal effects. A detailed analysis follows below.

#### Indicator weight analysis within the aviation logistics subsystem.

As shown in Table 4, the local government expenditures on civil aviation development funds (0.1975) is the most influential indicator in the aviation logistics subsystem. This is because government intervention and financial investment are crucial for the development and enhancement of infrastructure, services, and overall growth within the subsystem. A higher allocation of funds directly supports the improvement of civil aviation facilities, boosts operational capacity, and drives the expansion of logistics capabilities. These factors significantly impact the effectiveness and efficiency of the aviation logistics system, making this indicator the most influential. This highlights the subsystem’s reliance on financial support and regulatory frameworks.

However, the civil aviation flight route miles (0.0605) and civil aviation cargo throughput (0.0635) carry the lowest weight, suggesting that while flight routes and cargo throughput are essential for the operational scale of aviation logistics, they do not guarantee improved performance on their own. For instance, merely increasing the number of flight routes or the volume of cargo handled does not automatically enhance efficiency, speed, or cost-effectiveness. These metrics become meaningful only when supported by other factors, such as well-organized operations and systems that can effectively manage the increased complexity that comes with expanded routes or greater cargo volumes.

A notable observation in the demand aspect of the aviation logistics subsystem is the consistently lower weights assigned to cargo transport indicators compared to passenger transport. This is likely due to the higher direct impact and greater demand elasticity associated with passenger transport. While aviation cargo has traditionally been viewed as a secondary activity [45], its significance should not be underestimated. There exists a complex interdependence between passenger and cargo operations, both in terms of revenue generation and operational complexity. Therefore, adopting a comprehensive approach to the airline business model, one that integrates both passenger and cargo operations, is crucial for ensuring sustainable development [46].

#### Indicator weight analysis within the regional economic subsystem.

In the regional economic subsystem, GDP (0.1383) stands out as the most significant indicator, serving as a fundamental measure of economic performance. A higher GDP typically indicates a more developed economy, offering greater opportunities for investment, business activity, and employment. Consequently, the impact of GDP on the level of regional economic development is both critical and direct. The proportion of investment in environmental pollution control relative to GDP (0.1289) is also noteworthy, as it establishes a direct connection between economic development and environmental sustainability. This highlights the increasing importance of environmental considerations in the economic development of Sichuan Province, especially in the context of rapid industrialization. Given that aviation logistics significantly contributes to environmental degradation, the urgency for investment in pollution control becomes even more pronounced [47]. This emphasizes the need for a coordinated approach to the development of both aviation logistics and environmental sustainability.

However, the relatively low weight assigned to the elasticity coefficients of energy consumption (0.0826) is primarily due to its indirect impact on regional economic vitality. As energy efficiency improves and cleaner energy sources are adopted, its direct influence on economic performance diminishes. Additionally, the decoupling of economic growth from energy consumption reduces the relevance of these coefficients in driving economic vitality [48], which further lessens the weight of these indicators.

In conclusion, the growth of Sichuan Province’s aviation logistics sector significantly depends on government support, including financial investment and policy initiatives. Additionally, to achieve high-quality and sustainable regional economic development, GDP is crucial in the short term. Simultaneously, pollution control measures must be emphasized to alleviate the negative environmental impacts that could obstruct long-term economic advancement.

### AL&RE coupling coordination degree analysis

Based on the indicator weights within each subsystem, this study further investigates the coupling and coordinated development of AL&RE through CCDM. By applying the established formulas and utilizing raw data from Sichuan Province, the results are displayed in Table 5.

**Table 5:** System development level, CD, CI and CCD between the AL&RE subsystems.

Year	System development level U_1_	System development level U_2_	CD	CI	CCD	Coordination Level
2013	0.0393	0.2495	0.6857	0.1444	0.3146	Mildly dysfunctional
2014	0.2036	0.3481	0.9651	0.2758	0.5159	Barely coordinated
2015	0.3529	0.3590	1.0000	0.3560	0.5966	Barely coordinated
2016	0.3606	0.3919	0.9991	0.3763	0.6131	Elementary coordination
2017	0.5169	0.4816	0.9994	0.4992	0.7063	Intermediate coordination
2018	0.8211	0.4716	0.9628	0.6464	0.7888	Intermediate coordination
2019	0.6493	0.5368	0.9955	0.5930	0.7684	Intermediate coordination
2020	0.3916	0.6663	0.9657	0.5290	0.7147	Intermediate coordination
2021	0.5307	0.6427	0.9954	0.5867	0.7642	Intermediate coordination
2022	0.3120	0.8013	0.8982	0.5566	0.7071	Intermediate coordination
2023	0.7578	0.7207	0.9997	0.7392	0.8597	Good coordination

**Fig 3:**
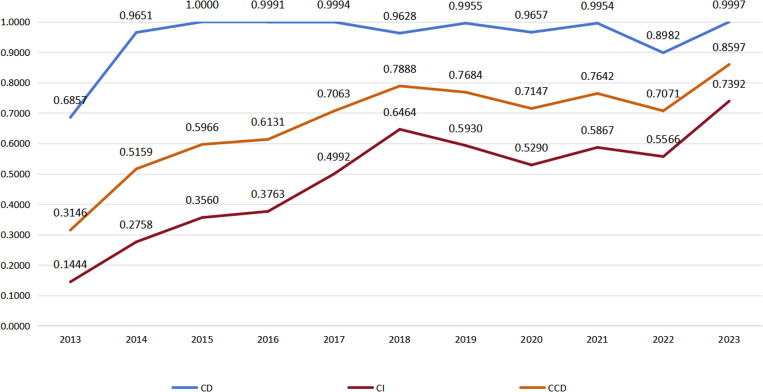
Trends in the AL&RE system coupling and coordination change.

To enhance the observation of changes in the CD, CI, and CCD of AL&RE system, this study produced the trend line graph presented in Fig 3.

As illustrated in Table 5 and Fig 3, the CD between the AL&RE subsystems significantly increased from 2013 to 2014, exceeding 0.9, and remained relatively stable thereafter, generally staying above this threshold. This indicates a strong interaction between the two subsystems, suggesting a close interconnection in the development of AL&RE in Sichuan Province. The robust coupling points to considerable potential for mutual growth, which aligns with the real-world scenario where Sichuan’s economy is heavily dependent on the aviation logistics sector.

The development trends of the CI and CCD follow similar patterns, both displaying a fluctuating upward trajectory. From 2013 to 2018, both the AL&RE subsystems exhibited relatively steady growth. This balanced development fostered enhanced coordination between the two subsystems, as reflected in the rising CI and CCD. Additionally, these improvements in coordination sustained to the continued positive development of both subsystems.

Between 2018 and 2022, however, coordination among the subsystems began to experience a gradual decline due to internal developmental imbalances. During this period, the aviation logistics subsystem faced a general downturn, despite the overall upward trend in the regional economy, both of which were accompanied by volatility. This divergence resulted in a pronounced disparity in the development levels of the two subsystems. This imbalance can largely be attributed to external shocks, such as the global economic slowdown, which reduced international trade volumes and, consequently, diminished the demand for aviation logistics services. Additionally, the COVID-19 pandemic, along with widespread lockdowns and travel restrictions, further disrupted air transportation, decreasing transport efficiency and disturbing global supply chains [49]. While the regional economy was also affected by these external factors, its diversified industrial structure and robust policy regulation helped mitigate the negative impacts, leading to an upward trend despite the volatility [50]. In contrast, the aviation logistics sector, being more sensitive to external changes, experienced greater volatility and a decline in its development level. These external shocks, including economic, health, and other global factors, contributed to imbalances in the development between subsystems, thereby hindering their coordinated growth.

Between 2022 and 2023, as the global economy began recovering from the COVID-19 pandemic, the aviation logistics sector experienced enhancements in transportation capacity and service levels, resulting in a swift recovery in its development level. The narrowing gap in development levels between the two subsystems encouraged closer coordination, as evidenced by the increased CI and CCD.

In conclusion, while the coupling between AL&RE remains strong, this does not necessarily imply high coordination. A high degree of coupling can coexist with internal imbalances or short-term mismatches in system adaptability. These imbalances are primarily driven by external disturbances impacting the subsystems. This highlights the detrimental effect of the uneven development of the AL&RE subsystems on their coordinated growth, while also underscoring the susceptibility of the coupling coordination between AL&RE to external influences. It stresses the necessity for adaptive strategies to improve coordination. Maintaining the stability and balanced development of both the AL&RE subsystems, along with enhancing forecasting and response capabilities to external shocks, is vital for boosting the resilience of both subsystems and ensuring their seamless coordination.

### Grey relational analysis between indicators and system coordination

Based on the coupling coordination results and the normalized indicator values, the grey relational degree was calculated to quantify the significance of each indicator in the coordinated development of the system, as shown in Table 6. The specific values obtained during the calculation process can be found in S4 Table.

**Table 6:** Grey relational degree between measurement indicators and CCD.

Measurement indicators	Relational degree	Rank
Civil aviation cargo throughput	0.9332	2
Civil aviation passenger throughput	0.9170	7
Cargo turnover of civil aviation	0.9471	1
Passenger turnover of civil aviation	0.8932	12
The volume of cargo transported by civil aviation	0.8978	11
The volume of passengers transported by civil aviation	0.9147	8
Number of employees in the aviation transport industry	0.8915	13
Civil aviation take-off and landing sorties	0.9332	2
Civil aviation flight route miles	0.9253	5
Local government expenditures on civil aviation development funds	0.7150	19
GDP	0.9015	10
Per capita national income	0.9040	9
Value added of tertiary industry as a proportion of GDP	0.9312	4
Total retail sales of consumer goods	0.9179	6
Total foreign trade import	0.8281	14
Total foreign trade exports	0.8006	15
Elasticity coefficient of energy consumption	0.7613	17
Elasticity coefficient of electricity consumption	0.7375	18
The proportion of investment in environmental pollution control relative to GDP	0.7787	16

As shown in Table 6, the selected indicators in this study demonstrate a strong grey relational degree with the coupling coordination between AL&RE. Specifically, 11 indicators have a relational degree exceeding 0.9, while 5 indicators surpass 0.8. This indicates that the indicator system developed in this study is both robust and effective in capturing the coupling and coordination between AL&RE.

Among the indicators, cargo turnover in civil aviation, civil aviation cargo throughput, and civil aviation take-off and landing sorties show high relational degrees, highlighting their significant impact on coupling coordination. Cargo turnover and throughput reflect the efficiency and capacity of the aviation freight network. An efficient freight network can enhance connectivity between regional products and businesses and external markets, accelerate economic circulation, and thereby stimulate regional economic development. Take-off and landing sorties, on the other hand, reflect the density and frequency of air transport, also influencing the efficiency of aviation logistics. Although they do not directly determine changes in logistics volume, they encourage positive interaction between the transportation activities of aviation logistics and regional economic development, promoting frequent economic activities. These factors create a conduit linking AL&RE, helping to transform the development of aviation logistics into economic outcomes and promoting the synergistic development of both.

In contrast, the grey relational degree of expenditure on local government civil aviation development funds is the lowest, indicating that while government and financial support are essential for the development of aviation logistics, their immediate impact on the coupling and coordination between AL&RE is limited. This suggests that such support does not necessarily lead to coordinated development or enhanced connectivity between AL&RE. While government investment in infrastructure can significantly impact and greatly support the development of aviation logistics, transforming these changes into tangible economic growth requires supportive policies and adaptation to market demand. If market demand is insufficient or industries cannot adapt to these changes, the improvement in aviation logistics will not directly result in rapid regional economic growth. Therefore, the impact on the synergistic development of AL&RE is relatively limited.

In summary, there exists a notable discrepancy between the key indicators that influence the development of the AL&RE subsystems and those that affect their coordination. For example, indicators such as cargo turnover and throughput have a relatively minor impact on the direct development of the aviation logistics subsystem. However, they play a more significant role in influencing the coupling and coordination between AL&RE. This is because, in the aviation logistics subsystem, these factors need to interact with other complementary elements to yield a substantial improvement. In contrast, coordination requires a more complex system integration that extends beyond individual metrics. Although the impact of these indicators on the direct development of aviation logistics is not immediately apparent, they can strengthen the interconnection of the aviation logistics subsystem with broader economic factors such as trade, industry, and economic activity. This explains why they score highly on the relational degree.

Thus, it is clear that the interaction between AL&RE is neither linear nor directly proportional. Even substantial improvements in one subsystem may not lead to effective coordination between AL&RE. Achieving high-level coordinated development between AL&RE requires, on one hand, a focus on the factors that significantly influence their coordination. On the other hand, attention must also be given to the simultaneous development of both subsystems to maintain balance and ensure effective coordination in their growth.

### Policy recommendations

Based on the analysis of key indicators influencing AL&RE development and their coupling coordination, the following policy recommendations are proposed for Sichuan Province:

(1) Enhancing the development of AL&RE subsystems: Given the significant role of local government expenditures on civil aviation development funds, Sichuan should continue to increase financial support for aviation logistics, particularly in infrastructure development, technological innovation, and policy formulation. Investments should extend beyond physical infrastructure to encompass supporting policies, talent acquisition, and technological research and development. In terms of regional economic development, GDP is the most direct factor influencing economic growth. However, environmental protection is essential for the long-term sustainability of economic development. Therefore, the government should prioritize environmental protection as a strategic long-term goal while generating economic returns by implementing stricter environmental policies and increasing investments in clean energy and eco-friendly technologies.
(2) Strengthening coordination: Sichuan should formulate policies that balance both short- and long-term objectives in AL&RE coordination. In the short term, efforts should concentrate on enhancing cargo turnover, cargo throughput, and civil aviation take-off and landing sorties to improve system coordination. This can be accomplished by streamlining customs procedures and implementing advanced logistics technologies to optimize cargo flow and minimize delays. Furthermore, investing in smart logistics systems and data-sharing platforms will enhance cargo management, tracking, and scheduling, thereby facilitating smoother operations. However, while these short-term measures may tackle immediate coordination challenges, they may not fully address the long-term requirements. Long-term policies should focus on resolving broader systemic challenges. To this end, long-term strategies should prioritize the optimization of both subsystems, while ensuring their balanced development. Additionally, regular assessments of development progress and coordination levels are essential to inform necessary policy adjustments, ensuring that the policies remain relevant and effective over time.
(3) Enhancing resilience to external shocks: Sichuan should bolster its capacity to forecast and respond to external uncertainties, such as global economic fluctuations and pandemics, which significantly impact the development of both AL&RE subsystems. Strengthening risk management strategies, improving supply chain resilience, and enhancing transportation flexibility and emergency response capabilities will help mitigate the adverse effects of external shocks, ensuring the stability and coordination of both subsystems. Specifically, this can be achieved by implementing advanced forecasting tools and risk assessment models to monitor potential disruptions, such as economic downturns, natural disasters, or health crises. Furthermore, supply chain resilience can be enhanced by encouraging companies to collaborate with multiple suppliers from diverse geographic regions, thereby reducing dependence on any single source. To improve transportation flexibility, investment in multi-modal logistics systems that integrate aviation, rail, and road transport is essential, enabling rapid shifts between modes in the event of disruptions. Finally, enhancing emergency response capabilities requires ensuring that critical logistics systems, such as digital platforms for tracking and management, are equipped with reliable backup systems to maintain functionality during system failures.

To translate these recommendations into actionable policies, Sichuan Province must establish a comprehensive implementation framework that ensures effective collaboration among government authorities, aviation companies, and logistics providers while also addressing legal and regulatory issues. A critical step in this process is developing a balanced incentive structure that aligns the interests of all stakeholders. This could involve creating a transparent profit-sharing mechanism to guarantee a fair distribution of financial benefits derived from investments in aviation logistics infrastructure, technological innovations, and environmental initiatives. Public-private partnerships (PPPs) can play a pivotal role in pooling resources and expertise, fostering collaboration while ensuring accountability and transparency.

From a legal and regulatory perspective, it is vital to develop clear, enforceable guidelines governing the allocation of government funds, infrastructure development, and the implementation of environmental policies. These guidelines should include mechanisms to ensure that aviation and logistics companies comply with environmental regulations, supported by incentives such as tax breaks or subsidies for sustainable practices, while penalties are imposed for non-compliance. Additionally, in the event of disruptions caused by external shocks, establishing a legal framework for risk-sharing is essential, where both businesses and the government bear the financial burden arising from global economic downturns, pandemics, or natural disasters.

By addressing these aspects, Sichuan can effectively transform its strategic recommendations into tangible outcomes, ensuring long-term sustainability, stability, and growth within the aviation logistics sector.

## Conclusions and outlook

This study builds upon existing research by conceptualizing AL&RE as a composite system and elevating their interaction to a coupled level. It establishes a coupling mechanism between AL&RE, analyzing the interactions and the coupling between them and their internal variables. Based on this, measurement indicator frameworks for the two subsystems are constructed, and the CCDM is applied to assess the coupling coordination level between them. Additionally, the GRA is integrated with the CCDM to identify critical factors influencing this coupling coordination. Empirical research is conducted using real data from Sichuan Province spanning 2013–2023. The main findings are as follows:

(1) The benefits associated with each indicator differ in the development of the subsystems. The most critical factor for the development of aviation logistics is the local government expenditures on civil aviation development funds, while the most critical factor for regional economic development is the GDP and the proportion of investment in environmental pollution control relative to GDP.
(2) The degree of coupling between AL&RE in Sichuan Province is extremely high, indicating a strong relationship and substantial potential for mutual enhancement. The overall coupling coordination level shows an upward trend, with occasional declines during specific periods. These declines are primarily attributed to the uneven development of AL&RE, influenced by external factors, which highlights the system’s coupling coordination vulnerability.
(3) The grey relational degree between the measurement indicators of AL&RE and their coupling coordination level is high, indicating that the selected indicators are representative. Furthermore, the factors most strongly associated with system coupling coordination are the cargo turnover of civil aviation, civil aviation cargo throughput, and civil aviation take-off and landing sorties, emphasizing their significance as critical factors in promoting the coordinated development of AL&RE.
(4) The inconsistency between the critical factors influencing the development of subsystems and coupling coordination highlights the nonlinear characteristics of the AL&RE composite system. This indicates that the coupling coordination between AL&RE does not adhere to a simple linear relationship. These system factors may interact in complex ways, leading to fluctuations or shifts in the overall coordination of AL&RE development.

However, this study has certain limitations. Firstly, the analysis of coupling coordination between AL&RE relies mainly on a static framework, which limits the understanding of their dynamic interactions. These subsystems are governed by nonlinear relationships, where the behavior of one subsystem can affect the other in a non-proportional and unpredictable manner. Therefore, the current static approach may not sufficiently capture the complexity of feedback loops. Furthermore, the existing measurement indicator systems for assessing AL&RE have limitations. While the current indicators offer valuable insights, they do not encompass all potential factors that could significantly impact the development and coordination of these two subsystems. Additional indicators, such as technological advancements and policy changes, have not been fully integrated into the evaluation framework, restricting its ability to provide a comprehensive assessment of coupling coordination. Future studies could adopt a dynamic modeling approach to better capture the evolving interactions and nonlinear relationships between AL&RE. Moreover, integrating additional indicators, such as technological progress and policy changes, would enhance the comprehensiveness of the measurement indicator systems, offering a more holistic understanding of the coupling coordination between these subsystems.

## Supporting information

S1 Table Raw data of the regional economy subsystem in Sichuan Province. (DOCX)

S2 Table Raw data of the aviation logistics subsystem in Sichuan Province. (DOCX)

S3 Table Normalized results of the measurement indicators. (DOCX)

S4 Table Calculation results for the grey relational coefficient of the sequence indicators. (DOCX)
