# Supplementary material for: Analyzing the coupling coordination between aviation logistics and the regional economy: Identifying coupling mechanisms and critical influencing factors
Source: PLoS One. 2025 May 9;20(5):e0323111. doi: 10.1371/journal.pone.0323111 (PMC12064044; doi:10.1371/journal.pone.0323111)
Supplement: S1 Table — (DOCX) [file pone.0323111.s001.docx]

**S1 Table. Raw data of the aviation logistics subsystem in Sichuan Province.**

| Indicator\Year | 2013 | 2014 | 2015 | 2016 | 2017 | 2018 | 2019 | 2020 | 2021 | 2022 | 2023 |
| --- | --- | --- | --- | --- | --- | --- | --- | --- | --- | --- | --- |
| Civil aviation cargo throughput (Unit: 10,000 tons) | 51.7 | 56.2 | 57.4 | 63.2 | 66.2 | 68 | 69.9 | 64.6 | 68 | 63 | 71.32 |
| Civil aviation passenger throughput (Unit: 100 million people) | 0.38 | 0.43 | 0.49 | 0.53 | 0.58 | 0.61 | 0.67 | 0.5 | 0.56 | 0.38 | 0.58 |
| Cargo turnover of civil aviation (Unit: 100 million ton-kilometers) | 8 | 8.8 | 9.7 | 10.5 | 11.4 | 12.1 | 12.8 | 12.8 | 14.5 | 13.1 | 14.3 |
| Passenger turnover of civil aviation (Unit: 100 million passenger-kilometers) | 543 | 628 | 717 | 783.68 | 856 | 954.7 | 1106.8 | 657.8 | 721.3 | 464.1 | 1105 |
| The volume of cargo transported by civil aviation (Unit: 10,000 tons) | 41 | 44.6 | 67 | 60 | 61 | 64.3 | 59 | 53 | 57 | 45 | 55.9 |
| The volume of passengers transported by civil aviation (Unit: 100 million people) | 0.34 | 0.38 | 0.42 | 0.46 | 0.5 | 0.55 | 0.64 | 0.43 | 0.47 | 0.30 | 0.66 |
| Number of employees in the aviation transport industry (Unit: 1,000 people) | 35.328 | 41.694 | 42.815 | 36.664 | 38.394 | 51.954 | 35.845 | 37.44 | 40.536 | 41.834 | 46.40 |
| Civil aviation take-off and landing sorties (Unit: 10,000 sorties) | 51.2 | 55.4 | 55.7 | 57.4 | 60.4 | 63.3 | 69 | 63.3 | 73.7 | 72 | 74.9 |
| Civil aviation flight route miles (Unit: 10,000 kilometers) | 53.6 | 85.1 | 97.2 | 112.4 | 102.6 | 121.4 | 133.4 | 145.6 | 131.5 | 160.7 | 170.3 |
| Local government expenditures on civil aviation development funds (Unit: 10,000 RMB) | 46850 | 51992 | 62651 | 77863 | 284478 | 582649 | 226288 | 105720 | 92251 | 23891 | 200538 |
