# Supplementary material for: Analyzing the coupling coordination between aviation logistics and the regional economy: Identifying coupling mechanisms and critical influencing factors
Source: PLoS One. 2025 May 9;20(5):e0323111. doi: 10.1371/journal.pone.0323111 (PMC12064044; doi:10.1371/journal.pone.0323111)
Supplement: S2 Table — (DOCX) [file pone.0323111.s002.docx]

**S2 Table. Raw data of the regional economy subsystem in Sichuan Province.**

| Indicator\Year | 2013 | 2014 | 2015 | 2016 | 2017 | 2018 | 2019 | 2020 | 2021 | 2022 | 2023 |
| --- | --- | --- | --- | --- | --- | --- | --- | --- | --- | --- | --- |
| Gross domestic product (GDP) (Unit: 100 million RMB) | 26518 | 28891.3 | 30342 | 33138.5 | 37905.1 | 42902.1 | 46363.8 | 48501.6 | 53850.8 | 56749.8 | 60132.9 |
| Per capita national income (Unit: RMB) | 14231 | 15749 | 17221 | 18808 | 20580 | 22461 | 24703 | 26522 | 29080 | 30679 | 32251 |
| Total retail sales of consumer goods (%) | 40.9 | 42.5 | 44.4 | 47.6 | 50.4 | 52.3 | 52.5 | 52.4 | 52.5 | 52.2 | 47.1 |
| Total Retail Sales of Consumer Goods (Unit: 100 million RMB) | 11001.0 | 12393.0 | 13834.4 | 15519.7 | 17404.5 | 19340.8 | 21343.0 | 20824.9 | 24133.2 | 24104.6 | 26313.4 |
| Total foreign trade imports (Unit: 100 million USD) | 2264.1 | 2540.2 | 1133.8 | 1415.5 | 2067.4 | 2614.1 | 2873.6 | 3427.5 | 3804.9 | 3861.6 | 3920.9 |
| Total foreign trade exports (Unit: 100 million USD) | 4195.2 | 4485 | 2056.5 | 1847.6 | 2538.5 | 3332.7 | 3892.3 | 4654.3 | 5708.7 | 6215.1 | 5448 |
| Elasticity coefficient of energy consumption | 0.46 | 0.41 | 0.01 | 0.3 | 0.31 | 0.45 | 0.59 | 0.5 | 0.79 | -0.03 | 0.44 |
| Elasticity coefficient of electricity consumption | 0.65 | 0.42 | -0.26 | 0.56 | 0.62 | 1.45 | 0.97 | 2.45 | 1.72 | 1.69 | 2.16 |
| The proportion of investment in environmental pollution control relative to GDP (%) | 0.89 | 1.07 | 1.22 | 1.43 | 1.3 | 0.78 | 0.71 | 1.6 | 0.63 | 0.92 | 0.94 |
