# Supplementary material for: Analyzing the coupling coordination between aviation logistics and the regional economy: Identifying coupling mechanisms and critical influencing factors
Source: PLoS One. 2025 May 9;20(5):e0323111. doi: 10.1371/journal.pone.0323111 (PMC12064044; doi:10.1371/journal.pone.0323111)
Supplement: S3 Table — (DOCX) [file pone.0323111.s003.docx]

**S3 Table. Normalized results of the measurement indicators.**

| Indicator\Year | 2013 | 2014 | 2015 | 2016 | 2017 | 2018 | 2019 | 2020 | 2021 | 2022 | 2023 |
| --- | --- | --- | --- | --- | --- | --- | --- | --- | --- | --- | --- |
| Civil aviation cargo throughput | 0.0000 | 0.2294 | 0.2905 | 0.5861 | 0.7390 | 0.8308 | 0.9276 | 0.6575 | 0.8308 | 0.5759 | 1.0000 |
| Civil aviation passenger throughput | 0.0000 | 0.1724 | 0.3793 | 0.5172 | 0.6897 | 0.7931 | 1.0000 | 0.4138 | 0.6207 | 0.0000 | 0.6897 |
| Cargo turnover of civil aviation | 0.0000 | 0.1231 | 0.2615 | 0.3846 | 0.5231 | 0.6308 | 0.7385 | 0.7385 | 1.0000 | 0.7846 | 0.9692 |
| Passenger turnover of civil aviation | 0.1228 | 0.2550 | 0.3935 | 0.4972 | 0.6098 | 0.7633 | 1.0000 | 0.3014 | 0.4002 | 0.0000 | 0.9972 |
| The volume of cargo transported by civil aviation | 0.0000 | 0.1385 | 1.0000 | 0.7308 | 0.7692 | 0.8962 | 0.6923 | 0.4615 | 0.6154 | 0.1538 | 0.5731 |
| The volume of passengers transported by civil aviation | 0.1111 | 0.2222 | 0.3333 | 0.4444 | 0.5556 | 0.6944 | 0.9444 | 0.3611 | 0.4722 | 0.0000 | 1.0000 |
| Number of employees in the aviation transport industry | 0.0000 | 0.3829 | 0.4503 | 0.0804 | 0.1844 | 1.0000 | 0.0311 | 0.1270 | 0.3132 | 0.3913 | 0.6659 |
| Civil aviation take-off and landing sorties | 0.0000 | 0.1772 | 0.1899 | 0.2616 | 0.3882 | 0.5105 | 0.7511 | 0.5105 | 0.9494 | 0.8776 | 1.0000 |
| Civil aviation flight route miles | 0.0000 | 0.2699 | 0.3736 | 0.5039 | 0.4199 | 0.5810 | 0.6838 | 0.7883 | 0.6675 | 0.9177 | 1.0000 |
| Local government expenditures on civil aviation development funds | 0.0411 | 0.0503 | 0.0694 | 0.0966 | 0.4664 | 1.0000 | 0.3622 | 0.1464 | 0.1223 | 0.0000 | 0.3161 |
| Gross domestic product (GDP) | 0.0000 | 0.0706 | 0.1138 | 0.1970 | 0.3388 | 0.4874 | 0.5904 | 0.6540 | 0.8131 | 0.8994 | 1.0000 |
| Per capita national income | 0.0000 | 0.0842 | 0.1659 | 0.2540 | 0.3523 | 0.4567 | 0.5811 | 0.6821 | 0.8240 | 0.9128 | 1.0000 |
| Value added of tertiary industry as a proportion of GDP | 0.0000 | 0.1379 | 0.3017 | 0.5776 | 0.8190 | 0.9828 | 1.0000 | 0.9914 | 1.0000 | 0.9741 | 0.5345 |
| Total retail sales of consumer goods | 0.0000 | 0.0909 | 0.1850 | 0.2951 | 0.4182 | 0.5446 | 0.6754 | 0.6416 | 0.8576 | 0.8558 | 1.0000 |
| Total foreign trade imports | 0.4055 | 0.5046 | 0.0000 | 0.1011 | 0.3350 | 0.5311 | 0.6242 | 0.8230 | 0.9584 | 0.9787 | 1.0000 |
| Total foreign trade exports | 0.5375 | 0.6039 | 0.0478 | 0.0000 | 0.1582 | 0.3400 | 0.4682 | 0.6426 | 0.8841 | 1.0000 | 0.8244 |
| Elasticity coefficient of energy consumption | 0.4024 | 0.4634 | 0.9512 | 0.5976 | 0.5854 | 0.4146 | 0.2439 | 0.3537 | 0.0000 | 1.0000 | 0.4268 |
| Elasticity coefficient of electricity consumption | 0.6642 | 0.7491 | 1.0000 | 0.6974 | 0.6753 | 0.3690 | 0.5461 | 0.0000 | 0.2694 | 0.2804 | 0.1070 |
| The proportion of investment in environmental pollution control relative to GDP | 0.2680 | 0.4536 | 0.6082 | 0.8247 | 0.6907 | 0.1546 | 0.0825 | 1.0000 | 0.0000 | 0.2990 | 0.3196 |
