# Supplementary material for: Analyzing the coupling coordination between aviation logistics and the regional economy: Identifying coupling mechanisms and critical influencing factors
Source: PLoS One. 2025 May 9;20(5):e0323111. doi: 10.1371/journal.pone.0323111 (PMC12064044; doi:10.1371/journal.pone.0323111)
Supplement: S4 Table — (DOCX) [file pone.0323111.s004.docx]

**S4 Table. Calculation results for the grey relational coefficient of the sequence indicators.**

| Sequence | Indicator\Year | 2013 | 2014 | 2015 | 2016 | 2017 | 2018 | 2019 | 2020 | 2021 | 2022 | 2023 |
| --- | --- | --- | --- | --- | --- | --- | --- | --- | --- | --- | --- | --- |
| x_0_ | Coupling coordination degree (CCD) | 0.8909 | 0.9358 | 0.8785 | 0.9050 | 0.8876 | 0.8842 | 0.9614 | 0.9446 | 0.9083 | 0.8162 | 0.9045 |
| x_j_ | Gross domestic product (GDP) | 0.8950 | 0.9384 | 0.8999 | 0.9299 | 0.8884 | 0.8628 | 0.9474 | 0.9376 | 0.9117 | 0.8183 | 0.9148 |
|  | Per capita national income | 0.7719 | 0.9268 | 0.9871 | 0.9559 | 0.9869 | 0.9248 | 0.9494 | 0.9969 | 0.9540 | 0.9910 | 0.7980 |
|  | Value added of tertiary industry as a proportion of GDP | 0.9173 | 0.9206 | 0.8915 | 0.9354 | 0.9086 | 0.8954 | 0.9941 | 0.9710 | 0.8989 | 0.8473 | 0.9165 |
|  | Total retail sales of consumer goods | 0.7763 | 0.8873 | 0.7242 | 0.7592 | 0.8088 | 0.8519 | 0.9327 | 0.8695 | 0.8317 | 0.7764 | 0.8917 |
|  | Total foreign trade imports | 0.6869 | 0.7870 | 0.7658 | 0.7313 | 0.7452 | 0.7796 | 0.8726 | 0.9394 | 0.8227 | 0.7216 | 0.9540 |
|  | Total foreign trade exports | 0.6322 | 0.8105 | 0.5896 | 0.9028 | 0.8339 | 0.9949 | 0.7653 | 0.8456 | 0.5776 | 0.5226 | 0.8996 |
|  | Elasticity coefficient of energy consumption | 0.9252 | 0.7577 | 0.5256 | 0.7478 | 0.7108 | 0.9265 | 0.8118 | 0.5311 | 0.7681 | 0.7411 | 0.6663 |
|  | Elasticity coefficient of electricity consumption | 0.7668 | 0.8333 | 0.8207 | 0.7349 | 0.8712 | 0.7425 | 0.7267 | 0.7303 | 0.6979 | 0.8773 | 0.7642 |
|  | The proportion of investment in environmental pollution control relative to GDP | 0.7858 | 0.9202 | 0.9956 | 0.9451 | 0.9904 | 0.9204 | 0.9637 | 0.9614 | 0.9463 | 0.9513 | 0.8851 |
|  | Civil aviation cargo throughput | 0.8283 | 0.9597 | 0.9636 | 0.9259 | 0.9568 | 0.9989 | 0.9008 | 0.9237 | 0.9532 | 0.7937 | 0.8824 |
|  | Civil aviation passenger throughput | 0.8537 | 0.9906 | 0.9574 | 0.9911 | 0.9441 | 0.9007 | 0.9643 | 0.9794 | 0.9266 | 0.9514 | 0.9586 |
|  | Cargo turnover of civil aviation | 0.8466 | 0.9742 | 0.9789 | 0.9338 | 0.9675 | 0.9648 | 0.8201 | 0.8504 | 0.8550 | 0.7310 | 0.9031 |
|  | Passenger turnover of civil aviation | 0.8228 | 0.9757 | 0.7971 | 0.8831 | 0.9667 | 0.9898 | 0.9408 | 0.9210 | 0.9198 | 0.8379 | 0.8207 |
|  | The volume of cargo transported by civil aviation | 0.8315 | 0.9723 | 1.0000 | 0.9533 | 0.9947 | 0.9986 | 0.8534 | 0.8939 | 0.9013 | 0.7499 | 0.9125 |
|  | The volume of passengers transported by civil aviation | 0.7604 | 0.8348 | 0.8907 | 0.9880 | 0.9170 | 0.9335 | 0.8225 | 0.8932 | 0.8944 | 0.9771 | 0.8950 |
|  | Number of employees in the aviation transport industry | 0.7878 | 0.9261 | 0.9929 | 0.9946 | 0.9261 | 0.8753 | 0.9569 | 0.9497 | 0.9870 | 0.9429 | 0.9259 |
|  | Civil aviation take-off and landing sorties | 0.9857 | 0.9571 | 0.9430 | 0.9847 | 0.8647 | 0.8856 | 0.9772 | 0.8952 | 0.9698 | 0.8141 | 0.9017 |
|  | Civil aviation flight route miles | 0.8774 | 0.7371 | 0.7142 | 0.7444 | 0.6321 | 0.3344 | 0.8244 | 0.7546 | 0.6882 | 0.5782 | 0.9797 |
|  | Local government expenditures on civil aviation development funds | 0.8909 | 0.9358 | 0.8785 | 0.9050 | 0.8876 | 0.8842 | 0.9614 | 0.9446 | 0.9083 | 0.8162 | 0.9045 |
